# Effectiveness of a provider and patient-focused intervention to improve hypertension management and control in the primary health care setting in Cuba: a controlled before-after study

**DOI:** 10.1186/s12875-022-01959-6

**Published:** 2023-01-14

**Authors:** Esteban Londoño Agudelo, Tullia Battaglioli, Addys Díaz Piñera, Armando Rodríguez Salvá, Tom Smekens, Fernando Achiong Estupiñán, Isabel Carbonell García, Patrick Van der Stuyft

**Affiliations:** 1 Facultad Nacional de Salud Pública, Grupo de Epidemiología, Universidad de Antioquia, Calle 62 #52-59, Medellín, Colombia; 2 Faculty of Medicine and Health Sciences, Department of Public Health and Primary Care, Ghent University. Campus UZ-Ghent, Corneel Heymanslaan 10, 9000 Ghent, Belgium; 3 Department of Public Health, Institute of Tropical Medicine, St. Rochusstraat 43, 2000 Antwerp, Belgium; 4 Facultad de Medicina, Universidad CES, Calle 10A #22-04, Medellín, Colombia; 5 Centro de Epidemiología y Salud Ambiental, Instituto Nacional de Higiene, Epidemiología y Microbiología (INHEM), Infanta No. 1158 e/ Llinás y Clavel, Centro Habana, 10300 La Habana, Cuba; 6 Centro Provincial de Higiene y Epidemiología, Buena Vista esquina Milanés, Matanzas, Cuba; 7 Centro Provincial de Higiene y Epidemiología, Calle J No. 1 entre 1ra y 2da Reparto Sueño, Santiago de Cuba, Cuba

**Keywords:** Hypertension, Chronic diseases, Primary health care, Family practice, Implementation research, Latin America, Cuba

## Abstract

**Background:**

Implementation research to improve hypertension control is scarce in Latin America. We assessed the effectiveness of an intervention aimed at primary care practitioners and hypertensive patients in a setting that provides integrated care through an accessible network of family practices.

**Methods:**

We conducted in Cardenas and Santiago, Cuba, a controlled before-after study in 122 family practices, which are staffed with a doctor and a nurse. The intervention comprised a control arm (usual care), an arm with a component targeting providers (hypertension management workshops), and an arm with, on top of the latter, a component targeting patients (hypertension schools). To evaluate the effect, we undertook a baseline survey before the intervention and an endline survey sixteen months after its start. In each survey, we randomly included 1400 hypertensive patients. Controlled hypertension, defined as a mean systolic and diastolic blood pressure below 140 and 90 mmHg, respectively, was the primary endpoint assessed. We performed linear and logistic regression with a Generalized Estimating Equations approach to determine if the proportion of patients with controlled hypertension changed following the intervention.

**Results:**

Seventy-three doctors, including substitutes, and 54 nurses from the 61 intervention family practices attended the provider workshops, and 3308 patients −51.6% of the eligible ones- participated in the hypertension schools. Adherence to anti-hypertensive medication improved from 42% at baseline to 63% at the endline in the intervention arms. Under the provider intervention, the proportion of patients with controlled hypertension increased by 18.9%, from 48.7% at baseline to 67.6% at endline. However, adding the component that targeted hypertensive patients did not augment the effect. Compared to patients in the control arm, the adjusted OR of having controlled hypertension was 2.36 (95% CI, 1.73–3.22) in the provider and 2.00 (95% CI, 1.68–2.37) in the provider plus patient intervention arm.

**Conclusions:**

The intervention’s patient component remains to be fine-tuned. Still, we demonstrate that it is feasible to substantially improve hypertension outcomes by intervention at the primary care level, despite an already relatively high control rate.

## Background

About 17.8 million people die from cardiovascular diseases (CVDs) each year, 31% of all deaths worldwide [1], and more than 75% of those deaths occur in low- and middle-income countries (LMICs) [2]. Uncontrolled hypertension, the main modifiable risk factor for CVDs, is associated with over 10 million avoidable deaths annually [3]. Almost three-quarters of people with hypertension (650 million people) live in LMICs [4], where 88% of the hypertension-attributable mortality takes place [5]. In Latin America and the Caribbean, hypertension affects up to 40% of the adult population [6, 7], and CVDs annually lead to 1.8 million deaths in the region [8, 9].

The benefits of lowering blood pressure (BP) to prevent CVDs are well established [10–14]. Notwithstanding, in a recent cross-sectional study on the management of hypertension in 44 LMICs [15], only 39.2% of individuals with hypertension were aware of their condition, 29.9% were being treated, and 10.3% had achieved BP control. A 2016 review including studies from 90 countries worldwide reported 26.3% hypertension control among patients aware of their condition in the represented LMICs, against 50.4% in the included high-income ones [16]. However, hypertension care and control figures show substantial variability among LMICs, corroborating the need to provide evidence on the contribution of risk factors, management strategies, and service delivery in their specific sociocultural and health care systems contexts [17]. Apart from the population’s possible low disease awareness and limited access to health care, most LMICs’ health systems are designed to provide acute curative care [18, 19]. Hence, chronic care is often reduced to fragmented and expensive disease management [20, 21], characterized by a lack of preventive approaches and oriented toward treating complications and acute exacerbations at the referral level [22, 23].

Yet, Primary Health Care (PHC) should play a key role in preventing and managing chronic conditions [22–26], also by boosting the quality of the provider-patient interaction. Well-tuned provider-patient communication and shared decision-making have been associated with better adherence to anti-hypertensive treatment and improved self-care management in diverse countries and populations [27, 28], including low-income black hypertensive patients in the United States [29, 30], patients attending PHC services in Latin America [31, 32] and patients treated in specialist services in Europe [33]. In addition, also provider-patient communication that addresses patients’ sociodemographic circumstances or promotes social support is instrumental in enhancing medication adherence [34, 35].

International initiatives such as the Standardized Hypertension Treatment and Prevention Project [19], developed by the Pan American Health Organization and the United States Centers for Disease Control and Prevention, have identified six essential components for intervention to improve hypertension control: guideline-based standardized treatment protocols; access to essential medicines and technology; health information systems for cohort monitoring and evaluation; patient empowerment; team-based care; and community engagement. The Global Hearts Initiative [36], led by the World Health Organization, stresses the need to strengthen health systems at the primary care level. The Initiative’s technical package addresses the four main risk factors for CVDs (tobacco use, physical inactivity, unhealthy diets, and harmful alcohol consumption). It further emphasizes risk-based management, appropriate referral, improved service delivery by task-sharing, and robust clinical monitoring. Interventions along the above lines, directed at the community, patients, and health care providers, have undoubtedly the potential to improve outcomes for people with chronic conditions in general and hypertension in particular. Still, there is a lack of evidence on their effectiveness from research conducted in LMICs [37]. Studies in Mexico [38, 39], Argentina [40], and Peru [41] are noteworthy exceptions in Latin America and the Caribbean.

In Cuba, a middle-income country, chronic conditions and CVDs account for 84 and 36% of the overall mortality, respectively [42]. Hypertension prevalence, in turn, was estimated at 30.9% in the population above 15 years in the most recent nationwide survey [43]. In contrast to the majority of fragmented Latin American health systems, the Cuban national system provides integrated care through an accessible network of family practices. It is internationally recognized for being well-organized, based on a PHC approach, and enjoying favorable health indicators [44–46]. In recent decades, the Cuban health system has shown remarkable hypertension control figures at the population level, similar to those reported by the United States and Canada [47]. It was signaled as an early example of significant success in controlling hypertension [48] and, at the same time, put forward as an appropriate setting to study the potential of resource-constrained health systems in improving hypertension treatment and control [49]. Indeed, the Cuban health system still faces significant challenges in comprehensively addressing non-communicable disease prevention and control. Despite free and universal health services, there are utilization and supply gaps, and there is scope for tweaking the quality of care [47, 50].

Our 2012 study amongst individuals with diagnosed hypertension in the municipalities of Cardenas and Santiago documented that 91% were on pharmacological treatment but only 58% had controlled hypertension [25]. Appraisal of the population’s representations regarding hypertension [51] documented unsatisfactory knowledge of hypertension management, self-care requirements, and potential complications. Audits of family doctors’ clinical records and assessment of the quality of the provided care [52] uncovered significant gaps in the PHC staff’s clinical performance and poor adherence to the national guidelines for hypertension management clinical inertia, and inadequate prescription of anti-hypertensive drugs. Based on this formative research, we set up an intervention to reduce the hypertension control gap, targeting primary care practitioners and hypertensive patients under their care. For the former, we focused on increasing compliance with evidence-based standardized guidelines, enhancing clinical skills, and improving the quality of the provider-patient interaction. For patients, the intervention primarily aimed to augment their awareness of the need for preventing complications, foster self-care skills, and improve hypertension treatment adherence. The present paper’s primary objective is to assess the effectiveness of the intervention in increasing the proportion of patients with controlled hypertension.

## Methods

### Design

We conducted a quasi-experimental controlled before-after study. We performed a baseline cross-sectional hypertension control assessment from February to December 2012 [25]. The intervention started in January 2013 and was actively implemented until January 2014. An intervention period of one year was deemed sufficient since most studies aimed at improving hypertension control could demonstrate results after one year or less [53]. We executed the endline survey between April and October 2014.

### Setting

The Cuban PHC system consists of Family Doctor and Nurse (FD&N) practices and policlinics. The entire population is registered with a FD&N practice, the first entry point to the health care system. Each practice comprises one doctor and one nurse, who share tasks towards providing holistic health care to their assigned population of around 1000 persons. The family doctor is the head of the team and is mainly in charge of curative clinical work, while the family nurse focuses on health promotion and prevention activities [54]. Doctors and nurses jointly perform home visits to conduct family and individual risk assessments, which guide preventive actions and define the schedules of follow-up consultations for each family member. Together with community representatives, they conduct an annual assessment of the local health situation and establish a plan for priority health-related activities.

Policlinics provide diagnostic and support services and specialized ambulatory care. One policlinic and about thirty FD&N practices compose a health area, covering around 30,000 inhabitants. Health areas are functionally organized into Basic Working Groups (BWGs) that assemble some 15 FD&N practices and an internist, pediatrician, obstetrician/gynecologist, psychologist, dentist, nurse supervisor, social worker, statistician, and hygiene and epidemiology technician from the policlinic [54]. The National Hypertension Program of the Ministry of Public Health sets and evaluates specific control policies and produces and updates the Cuban guidelines for hypertension prevention, diagnosis, and treatment [55].

### Study population and sampling

The study took place in the health areas Julio Antonio Echeverria (population 35,258; 2 BWGs and 28 FD&N practices) and Moncada (population 37,513; 2 BWGs; 33 FD&N practices) in the municipality of Cardenas (central Cuba; total population 142,369), and in the Grimau (population 41,284; 2 BWGs; 33 FD&N practices) and Finlay (population 28,885; 2 BWGs; 28 FD&N practices) health areas in Santiago municipality (east Cuba; total population 513,784). The municipalities were chosen based on the commitment of the local health authorities to facilitate carrying out the study and to collaborate in assuring high implementation quality of the intervention.

**Fig. 1:**
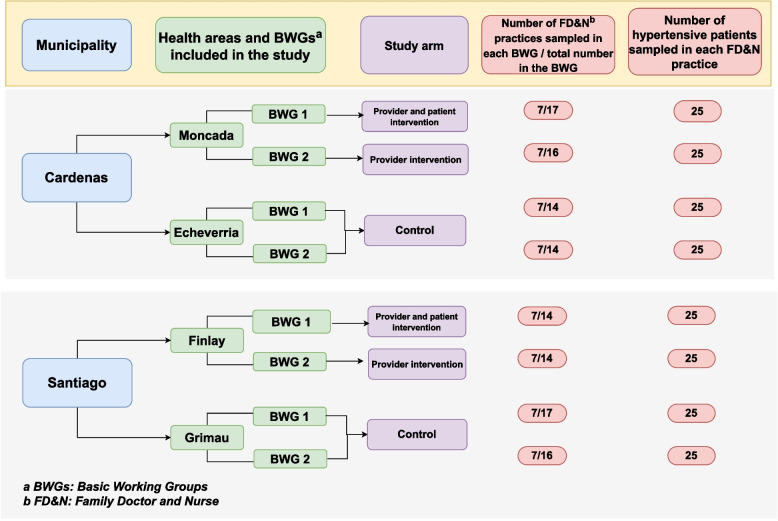
Intervention allocation and sampling procedure in each survey round. Cardenas and Santiago, Cuba, 2012–2014

We independently sampled participants for the baseline and endline surveys. The sample size for each survey was 1400 hypertensive patients. In each of the 2 BWGs of the 4 study health areas, we selected 7 FD&N practices by systematic random sampling from the list of existing BWG practices in the area, using a random starting point and the number of FD&N practices in the area/7 as sampling interval. We included 25 patients by simple random sampling from the hypertension register in each of these practices. Patients were eligible for inclusion if they were 18 years or older, had a confirmed diagnosis of hypertension documented in their medical records, and provided written informed consent to participate in the study. In the baseline survey, the sample size allowed to estimate the proportion of patients with controlled hypertension -anticipated being around 50%- with a confidence level of 90% and a precision of 5%, given an expected cluster effect of 2.5. For the endline survey, it provided 80% power at α 0.05 to detect a 15% increase in hypertension control in the intervention arms. Figure 1 depicts a graphical illustration of the study population, intervention allocation, and sampling procedure.

### Intervention

The intervention targeted FD&N and hypertensive patients under their care. In each municipality, the intervention was assigned to the study health area with the higher prevalence of uncontrolled hypertension in diagnosed patients: Finlay in Santiago and Moncada in Cardenas. In each of these areas, we implemented the health care provider component in both BWGs, and in one randomly selected BWG, we added the component targeting hypertensive patients. The second study health area in each municipality, Grimau and Echeverria, served as control. There, the National Hypertension Program continued to be implemented unabated, and patients received the usual care.

### Description of the intervention

#### Hypertension management workshops for family doctors and nurses

Together with the Provincial Technical Advisory Commissions on Hypertension and the policlinics’ management, the research team developed seven training sessions of two hours each for doctors and nurses of the family practices in both intervention arms. They focused on updating the knowledge and skills required for good quality diagnosis, treatment, and follow-up of hypertensive patients. Members of the Provincial Commission and internal medicine specialists from the policlinics delivered the workshops.

The training was designed as four interactive conferences and three practical sessions, each one week apart. The conferences focused on epidemiological aspects of hypertension, the Cuban surveillance system for non-communicable diseases, and various aspects of hypertension diagnosis and management (Panel). The practical training aimed at strengthening skills in correctly taking BP and anthropometric measurements and accurately recording clinical data.

Before attending the workshops, participants were invited to study the Cuban hypertension prevention and control guidelines. The training adopted a formative model for adult learning. A pre-test evaluated knowledge gaps and strengths on the subject. During the conferences, the attendees reflected upon and discussed the main aspects of the topics covered using interactive group techniques. The hands-on sessions relied on standardized protocols and demonstrations to develop specific clinical skills. Finally, the policlinics’ management conducted a summary and feedback session and applied a post-test to evaluate the participants’ progress.

#### Hypertension schools

The family nurses and policlinic staff from different disciplines conducted the Schools in the FD&N practices of the ‘Provider and Patient’ study arm. The Schools aimed to improve hypertensive patients’ understanding of their condition and foster self-reliance. They consisted of interactive sessions one week apart that received a maximum of twenty patients per cycle.

Patients with complications such as ischemic heart disease, stroke, and end-stage renal insufficiency or elderly patients with multi-pathology are primarily followed up by a multidisciplinary specialist team in the policlinics. Hence, given the focus of the intervention on strengthening the system’s performance at the FD&N practice level, the research team, in consultation with the Ministry of Health, decided to limit attendance to patients of age 20 to 70 with uncomplicated hypertension.

Attendees received a reminder phone call one day before each of the four regular School sessions, which lasted an hour and delivered critical messages adapted to the local customs. Subjects covered included self-care and modifiable factors such as healthy diets, physical activity, excessive alcohol consumption, and tobacco use. Patients also obtained information on abnormal BP values, the importance of periodical BP measurements, how uncontrolled hypertension affects health, and alarm signs for the most frequent cardiovascular complications. Moreover, they received education on the relevance of adherence to anti-hypertension treatment. At the end of each session, a physiotherapist briefly intervened on how to keep physically active and maintain a healthy weight. This was followed by thirty minutes of guided physical exercise. Additionally, a fifth closing session brought patients and their relatives together to share essential hypertension information and discuss household support.

**Table:** Panel. Target, components, and implementation strategy of the intervention to improve hypertension management and control. Cardenas and Santiago, Cuba, 2013

**Target **	**Description**
**Health care providers:** family doctors and nurses	**Hypertension management workshops** - 7 workshops of 2 hours each, spread over two months, followed by a theory and practice test - the first session started in January 2013; the second session, for substitute staff, started in September 2013 - facilitated by specialists of the policlinic and staff of the provincial Technical Advisory Commission on Hypertension - subjects: review of the Cuban hypertension control program risk factors for hypertension, complications of hypertension, primary and secondary prevention BP and anthropometric measurements, diagnosis of hypertension therapeutic inertia, pharmacological and non-pharmacological treatment, indications and contra-indications adherence to treatment, adverse effects of treatment, adapting treatments Information, Education, and Communication (IEC): essential elements to address during patient consultation for hypertension patient counseling and follow-up, proper medical record keeping
**Hypertensive patients:** 20 to 70 years old with essential uncomplicated hypertension and no major co-morbidities	**Hypertension schools** - 4 interactive sessions of 1 hour each with patients followed by 30 minutes of physical exercise, spread over four weeks, with a maximum of 20 participants - 1 additional session with patients and their family members to share essential information and discuss household support - first cycles started in March 2013 - facilitated by a multidisciplinary team of family doctors, nurses, psychologists, nutritionists, and physiotherapists directed by the BWG coordinator - subjects: BP control, prevent and identify complications of hypertension, alarm signs healthy lifestyles, healthy diets, food preparation need for physical exercise and adapted schemes importance of adherence to treatment and how to secure it

### Data collection and management

In both survey rounds, trained research staff visited the selected hypertensive patients at home for interviewing and BP, height, and weight measurements. If the person was absent, two further visits were made before choosing a replacement. All participants were interviewed using a closed questionnaire covering sociodemographic characteristics, lifestyle habits, past and current health problems, anti-hypertensive pharmacological treatment, and treatment adherence. To determine levels of adherence to pharmacological treatment, we applied the four-item medication adherence questionnaire by Morisky et al. [56]. BP was measured three times, from the right arm in a sitting posture, using a mercury manometer, following international recommendations for BP measurement in population surveys [57, 58]. Participants’ weight and height were recorded to the nearest 0.1 kg and 0.01 m.

To ensure data collection reliability and consistency, the local research staff received training in using the data collection tools and had to pass a standardized test on BP measurement. The data collection process was supervised and audited by central research staff from Havana. Data were double-entered in a Microsoft Access 2000 database with built-in filters and logical constraints to assess the completeness and accuracy of digitalization.

### Data analysis

We calculated the mean of the last two BP measurements and defined controlled hypertension as a mean systolic and diastolic BP lower than 140 and 90 mmHg, respectively. Patients with a body mass index ≥30 kg/m2 were classified as obese. Higher education was taken as the completion of at least secondary studies. We categorized skin color as white and non-white (comprising mestizo and black). Means/standard deviations (SD) and proportions/95% confidence intervals (95%CI) were calculated for continuous and categorical variables, respectively.

To estimate whether the proportion of patients having controlled versus uncontrolled hypertension had changed following the implementation of the intervention, we performed linear and logistic regression with a Generalized Estimating Equations (GEE) approach. The BWG, the level of intervention allocation, was taken as the clustering variable under an exchangeability correlation structure. The effect of the intervention was coded as the interaction of the study arm (intervention/control) with the survey round (baseline/endline). We report crude percentage point differences and odds ratios (OR) with 95%CI and adjusted OR after including other independent variables in the regression model and controlling for possible confounding factors. The latter were selected based on being associated with hypertension control and study arm in bivariate analysis or being considered of relevance a priori. We did not include the predictor of anti-hypertensive pharmacological treatment in the model for being on the causal pathway from the intervention to hypertension control. Moreover, the variable health area was not entered in favor of the BWG as the clustering variable.

## Results

All hypertensive patients that were successfully contacted consented to participate in the surveys. However, in the baseline survey, sixty-seven sampled patients (5%), mainly from the Echeverria health area, were not encountered at home after two repeat visits and were not replaced due to operational constraints. Hence, the total number of respondents was 1333. The endline survey included 1404 respondents. We excluded 4 cases with a missing value for education or obesity, leaving 2733 participants in the analyses (Table 1).

**Table 1:** General characteristics, hypertension treatment, and blood pressure parameters of 2733 registered hypertensive patients in the baseline and endline survey by study arm. Cardenas and Santiago, Cuba, 2012–2014

Variable (%) ^a^	Control arm		Intervention arms			
			Provider		Provider and Patient	
	Baseline	Endline	Baseline	Endline	Baseline	Endline
**Sex**						
Male	229 (36)	266 (38)	128 (37)	143 (40)	157 (45)	131 (38)
Female	406 (64)	433 (62)	221 (63)	211 (60)	191 (55)	217 (62)
**Age**						
< 65	402 (63)	407 (58)	188 (54)	203 (57)	217 (62)	183 (53)
≥ 65	233 (37)	292 (42)	161 (46)	151 (43)	131 (38)	165 (47)
**Ethnicity**						
Non-white	394 (62)	402 (58)	168 (48)	189 (53)	195 (56)	163 (47)
White	241 (38)	297 (42)	181 (52)	165 (47)	153 (44)	185 (53)
**Civil status**						
Single	267 (42)	280 (40)	153 (44)	153 (43)	151 (43)	153 (44)
Married / partner	368 (58)	419 (60)	196 (56)	201 (57)	197 (57)	195 (56)
**Post-primary Education**						
No	79 (12)	114 (16)	79 (23)	51 (14)	47 (14)	45 (13)
Yes	556 (88)	585 (84)	270 (77)	303 (86)	301 (86)	303 (87)
**Paid Job**						
No	365 (57)	404 (58)	239 (68)	159 (45)	206 (59)	154 (44)
Yes	270 (43)	295 (42)	110 (32)	195 (55)	142 (41)	194 (56)
**Obese**						
No	522 (82)	604 (86)	265 (76)	299 (84)	280 (80)	289 (83)
Yes	113 (18)	95 (14)	84 (24)	55 (16)	68 (20)	59 (17)
**Diabetes**						
No	537 (85)	560 (80)	295 (85)	292 (82)	293 (84)	277 (80)
Yes	98 (15)	139 (20)	54 (15)	62 (18)	55 (16)	71 (20)
**Coronary Heart Disease**						
No	569 (90)	548 (78)	284 (81)	294 (83)	294 (84)	291 (84)
Yes	66 (10)	151 (22)	65 (19)	60 (17)	54 (16)	57 (16)
**On anti-hypertensive pharmacological treatment**						
No	54 (9)	29 (4)	30 (9)	20 (6)	31 (9)	17 (5)
Yes, not adherent	280 (44)	310 (44)	174 (50)	117 (33)	167 (48)	103 (30)
Yes, adherent	301 (47)	360 (52)	145 (42)	217 (61)	150 (43)	228 (66)
**Blood pressure, mmHg (± SD)**^b^						
Systolic	129.6 (13.9)	131.0 (14.9)	132.0 (15.6)	128.6 (13.9)	128.8 (13.9)	126.1 (14.2)
Diastolic	82.0 (8.76)	83.4 (8.76)	84.5 (9.71)	82.0 (8.39)	83.6 (83.6)	80.3 (80.3)
**Total**	**635 (100)**	**699 (100)**	**349 (100)**	**354 (100)**	**348 (100)**	**348 (100)**

^a^ number (%) for categorical variables

^b^ mean (standard deviation) for numerical variables

The mean age (SD) of participants in the baseline and endline survey was 59.8 (14.0) and 60.9 (14.5) years, respectively. In both surveys, there were 1.6 times more women than men. Table 1 provides further details on the general characteristics of the included patients. Notably, most participants were on pharmacological anti-hypertensive treatment, but the percentage of non-adherence was high. In addition, the adherence increased substantially from baseline to endline in both intervention arms but not in the control arm.

Seventy-three family doctors from 61 intervention FD&N practices attended the provider workshops. This number includes staff present at the start and possible substitutes for family doctors posted elsewhere or participating in international missions who were trained in September 2013. Nurses were posted much more stably, but only 54 attended, mainly due to the workshops being organized outside working hours. Eventually, 3308 hypertensive patients in the ‘Provider and Patient’ study arm, 51.6% of the eligible ones, participated in the hypertension schools. The main reason for not attending was a conflict between the schedule of the sessions and patients’ labor obligations.

In Table 2, we present the crude intervention effect. In absolute terms, the difference in % endline minus % baseline hypertension control was 18.7% higher in the study’s intervention arms than in the control arm (*p* < 0.001). The odds of attaining hypertension control in the second survey round were more than twice higher (*p* < 0.001) in the intervention arms than in control.

**Table 2:** Crude effect of the intervention on hypertension control in registered hypertensive patients. Cardenas and Santiago, Cuba, 2012–2014

Study arm	Controlled hypertension			Intervention effect	
	Baseline	Endline	% difference	Difference of differences^a^	OR
	n/N (%; 95% CI)	n/N (%; 95% CI)	(95% CI)	(95% CI)	(95% CI)
Control	399/635 (62.5; 60.3–64.8)	423/699 (60.5; 55.8–65.1)	−2.4 (−6.5–1.6)	Reference	Reference
Provider intervention	170/349 (48.7; 45.3–52.1)	239/354 (67.6; 56.0–77.4)	18.8 (11.4–26.3)	21.3 (12.8–29.8)	2.43 (1.63–3.62)
Provider and patient intervention	203/348 (58.4; 55.7–61.0)	250/348 (71.8; 69.6–73.9)	13.5 (13.0–14.0)	16.0 (11.9–20.1)	2.02 (1.71–2.40)

All estimates and CIs are corrected for clustering in BWGs using GEE

*OR* Odds Ratio, *CI* Confidence Interval

^a^ coefficients of the “study arm by survey round” interaction term with no control for confounding (see text)

After controlling for potential confounding factors in multivariable logistic regression (Table 3), the odds of having attained controlled hypertension remained twice or higher in patients in the study’s intervention arms than in the control arm (study arm by survey round interaction terms; *p* < 0.001). The point estimate of the intervention effect was somewhat higher for the provider only as compared to the provider and patient intervention arm, but confidence intervals largely overlapped. In both municipalities, the intervention components were allocated to the health areas with the lower baseline hypertension control prevalence, which is reflected by the ORs below 1 under the study arm variable. The OR for the endline survey round is not significantly different from 1, indicating no baseline to endline change in the control arm. Considering the whole study population, having post-primary education, not being obese, and being of white ethnicity were independently and positively associated –be it rather weakly- with hypertension control.

**Table 3:** Adjusted intervention effect and predictors of controlled hypertension in registered hypertensive patients. Cardenas and Santiago, Cuba, 2012–2014

Predictor	Adjusted OR (95%CI)^a^
**Study arm**	
Control	Reference
Provider intervention	0.57 (0.50–0.65)
Provider and patient intervention	0.82 (0.70–0.95)
**Survey round**	
Baseline	Reference
Endline	0.90 (0.76–1.06)
**Intervention effect**^b^	
Control	Reference
Provider intervention	2.36 (1.73–3.22)
Provider and patient intervention	2.00 (1.68–2.37)
**Age**	
< 65	Reference
≥ 65	0.84 (0.73–0.97)
**Ethnicity**	
Non-white	Reference
White	1.45 (1.31–1.61)
**Post-primary education**	
No	Reference
Yes	1.47 (1.22.-1.78)
**Obesity**	
No	Reference
Yes	0.68 (0.56–0.86)

*OR* Odds Ratio, *CI* Confidence Interval

^a^ OR and 95% CI from a multivariable GEE model (see text)

^b^ OR from the coefficients of the “study arm by survey round” interaction term in the model

## Discussion

This controlled before-after study in 122 FD&N practices in two Cuban municipalities found that an intervention targeting PHC providers resulted in a substantially increased proportion of patients with controlled hypertension. However, adding to the provider component a behavioral intervention that targeted the hypertensive patients under their care did not augment the effect. In both intervention arms of the study, the adherence to anti-hypertensive pharmacological treatment improved markedly from baseline to endline, and the average patients’ systolic and diastolic BP decreased. After the intervention, the adjusted odds ratio of having controlled hypertension was twice higher in the study’s intervention arms than in the usual care arm.

Our findings can be compared with the results obtained in a one-year, uncontrolled HEARTS [59] intervention led by the Pan American Health Organization in the catchment area of a policlinic in the Matanzas province, Cuba [60]. It improved hypertension control in registered patients by almost 10%, from 59.3 to 68.4%. Averaging over both intervention arms, we attained close to a 20% difference of difference increase in the intervention arms of our controlled study. A multi-component cluster-randomized trial conducted in public primary care centers in Argentina [40] resulted, after 18 months, in a difference of difference of about 21% hypertension control increase in the intervention group compared to the usual care group. However, its baseline control prevalence was only 17% against over 50% in our study.

In a recent non-controlled patient-centered intervention conducted in three health centers in Lima, Peru [41], the use of BP lowering medication increased from 73 to 82% after 24 months. In our intervention, the vast majority of patients were already on pharmacological treatment at baseline, but the proportion with adequate adherence increased by around 20%. We hypothesize that the training of the FD&N practitioners on recommended clinical and treatment guidelines [61–63] enhanced prescribing of standardized anti-hypertensive treatment regimens, as was observed in a trial conducted in Nigeria and China [64]. At the same time, it should have improved their skills for interacting with and motivating patients, which could have contributed to boosting patients’ medication adherence.

A review of health care provider-led interventions on lifestyle modification in hypertensive patients in high-income countries [65] supports the idea that this approach can contribute to lowering elevated BP. Therefore, the apparent lack of increased hypertension control when adding the intervention component targeting hypertensive patients on top of our provider component is somewhat surprising. However, only around 52% of patients attended hypertension schools. Additionally, the serial small group organization of the schools delayed building up coverage. In contrast, in Costa Rica, a country similar to Cuba in terms of PHC approach and structure, a healthy lifestyle intervention to reduce the cardiovascular disease risk of hypertensive and diabetic patients resulted, after adjusting for confounding variables, in a significant average 8 and 5 mmHg reduction in systolic and diastolic BP, respectively, despite only 58% of the patients enrolled in the intervention group having attended at least one group education session [66].

A possible explanation for the lack of effect of our educational component is that we targeted patients with uncomplicated hypertension, possibly excluding the ones which might have benefitted more: the individuals at higher CVDs risk that require intensive and frequent lifestyle counseling [36], who were already under specialist care. On the other hand, in our hypertension schools, we relied on interactive educational group sessions, which are more effective than self-learning by reading and classic lectures as educational strategies for attaining behavioral change [67]. However, on the pathway between exposure to the intervention and hypertension control, lifestyle modifications are subject to underlying and interrelated behavioral, nutritional, social, and environmental drivers, and change remains uncertain [5].

Unsurprisingly, a Cochrane systematic review [53] found that isolated educational interventions unlikely lead to significant BP reductions. However, it also indicated that nurse-led care might have potential and that successful hypertension control trials were always multi-component and included, as critical elements, registration and regular follow-up of patients and a stepped approach to anti-hypertensive treatment. A more recent review [68] corroborates that multi-component strategies are the most effective, alongside team-based care, but notes that data from LMICs are sparse. Finally, it has also been pointed out that task sharing or shifting with non-physician health care workers in these countries may produce favorable effects on BP control [69].

Our study has some limitations. First, given the integrated nature of the Cuban health system and close social networks within the society at large, some cross-contamination between study arms might have occurred. However, the control (usual care) and intervention activities were allocated to administrative and operationally independent health areas and BWGs, which put up a barrier against it. Of note, contamination -if any- will have diluted the intervention effect. Second, the output of the different elements making up the intervention components was not measured separately, and we cannot sort out which ones may have contributed most to the outcome. Third, the inclusion of process evaluations has been encouraged for assessing multi-component interventions [37]. We controlled the delivery of the components of our intervention regarding content and targeted population but did not collect structured information on the process and fidelity of their implementation. This may hamper the interpretation of our results and compromise the ease of scaling up the intervention. Finally, from a subject matter point of view, the provider component somewhat neglected the training on cardiovascular risk assessment and the use of treatment algorithms.

The study also has clear strengths. First, it is among a few studies in LMICs [38–41, 70] that evaluate the effectiveness of a multi-component intervention to improve hypertension control by building on the potential of PHC. The Cuban public health system is homogeneous and provides universal access and integrated care through a dense nationwide PHC network. Our results should be replicable nationally but the intervention may not be transferable to other LMICs without adapting its components and activities to the local context and prevailing bottlenecks for improving hypertension control. On the other hand, given the poor control levels currently attained in many resource-constraints settings, the opportunities to pick low-hanging fruit may be considerable. Second, it was preceded by formative research [25, 51, 52] and developed with the active participation of the Hypertension Program of the Cuban Ministry of Health, local health system staff, and the communities belonging to the catchment areas of the involved FD&N practices -in line with recommendations to incorporate local communities as co-designers of interventions tailored to their needs [71]. Third, the intervention was carried out by existing PHC staff, minimizing the need to allocate additional resources and enhancing the potential for scaling up.

## Conclusions

We demonstrate that taking inspiration from the World Health Organization’s Hearts Initiative, it is feasible to substantially improve hypertension outcomes by intervention at the PHC level, even in a health care system that has already achieved a relatively high control rate. In preparation for scaling up the intervention to other health areas, further implementation research should tackle the challenges ahead: strengthening cardiovascular risk assessment and the use of simple treatment algorithms in the provider component, fine-tuning the behavioral change messages in the patient component, complementing the intervention with a community component targeting the public at large, and studying in-depth the implementation process.

## Data Availability

The data supporting this study’s findings are available from the Cuban National Institute of Hygiene, Epidemiology, and Microbiology (INHEM), according to legal procedures defined by the Cuban authorities. Data will be shared upon reasonable request to Dr. Armando Rodríguez Salvá (armando.rdguez@infomed.sld.cu) after signing a data access agreement with INHEM.

## Abbreviations

CVDs: Cardiovascular diseases
LMICs: Low- and middle-income countries
BP: Blood pressure
PHC: Primary health care
FD&N: Family doctor and nurse
BWG: Basic working group
GEE: Generalized estimating equations
